# Long-Term Effect of Anti-Vascular Endothelial Growth Factor (Anti-VEGF) Injections in Choroidal Neovascularization Secondary to Angioid Streaks

**DOI:** 10.1155/2022/3332421

**Published:** 2022-07-09

**Authors:** Sónia Torres-Costa, João Bernardes, Sofia Sousa Mano, Joana Medeiros-Pinto, Ana Carolina Abreu, Maria João Furtado, Rufino Silva, Carlos Marques-Neves, Fernando Falcão-Reis, Ângela Carneiro, Luísa Colaço, Manuel Falcão

**Affiliations:** ^1^Department of Ophthalmology, Centro Hospitalar Universitário de São João, Porto, Portugal; ^2^Department of Ophthalmology, Centro Hospitalar Universitário de Coimbra, Coimbra, Portugal; ^3^Faculty of Medicine, University of Coimbra (FMUC), Coimbra, Portugal; ^4^Department of Ophthalmology, Centro Hospitalar Universitário Lisboa Norte, Lisboa, Portugal; ^5^Department of Ophthalmology, Centro Hospitalar Universitário Do Porto, Porto, Portugal; ^6^Association for Innovation and Biomedical Research on Light and Image (AIBILI), Coimbra, Portugal; ^7^Ophthalmology Clinic, Faculdade de Medicina, Universidade de Lisboa, Lisboa, Portugal; ^8^Faculty of Medicine, University of Porto, Porto, Portugal; ^9^Department of Ophthalmology, Centro Hospitalar Fernando da Fonseca, Amadora, Portugal

## Abstract

**Purpose:**

This study aimed to evaluate the long-term effectiveness of intravitreal anti-vascular endothelial growth factor (VEGF) injections in the treatment of choroidal neovascularization (CNV) associated with angioid streaks.

**Methods:**

Multicenter retrospective cohort study, including eyes with CNV secondary to angioid streaks treated with anti-VEGF injections, were performed. Best-corrected visual acuity (BCVA) in ETDRS letters; qualitative and quantitative (foveal thickness) OCT parameters; anti-VEGF type; and number of injections were collected at baseline and at 3, 6, 12, 24, 36, 48, 60, and 72 months.

**Results:**

Thirty-nine eyes from 29 patients, 17 (58.6%) females, were included. The mean follow-up time was 69.4 ± 34.5 months. BCVA was 59.3 ± 23.3 letters at baseline and 63.7 ± 21.9 letters at 48 months. At 3 months, BCVA improved 6.9 ± 11.7 letters (*P*=0.003). Then, BCVA remained stable. The mean foveal thickness decreased from 343.3 ± 120.2 *μ*m at baseline to 268.3 ± 65.4 at 48 months (*P*=0.021). The mean number of injections was 4.6 ± 2.1 at 12 months, decreasing to 1.7 ± 2.4 injections between 36 and 48 months (*P*=0.093).

**Conclusion:**

This real-world study suggests that the functional and morphologic response to anti-VEGF therapy for CNV related to angioid streaks is generally satisfactory and maintained in the long term.

## 1. Introduction

Angioid streaks were first described as irregular red/brown lines that surround concentrically or radiate out of the optic nerve head [1, 2]. Currently, the estimated prevalence of angioid streaks is unknown. The onset of angioid streaks commonly lies between the second and fifth decades [3]. Pseudoxanthoma elasticum (PXE) is the most common associated disease [4]. Patients with angioid streaks are generally asymptomatic, unless the lesions extend towards the fovea or develop complications, such as macular choroidal neovascularization (CNV) [2, 4]. In fact, CNV is the most serious complication of angioid streaks, with an incidence of 72% to 86% [1].

Around the fourth to fifth decades of life, the visual acuity of untreated patients is less than 20/200 due to disciform scar formation and concomitant atrophy [2]. The management of CNV secondary to angioid streaks with intravitreal anti-vascular endothelial growth factor (VEGF) injections, irrespective of the molecule (i.e., bevacizumab, ranibizumab, or aflibercept), has shown superior functional and morphologic results compared with previous therapeutic options, such as photocoagulation laser and photodynamic therapy (PDT). Most literature on anti-VEGF treatment in CNV related to angioid streaks was based on single case reports, case series, or short-term cohorts [2, 4–11]. Recently, long-term retrospective and prospective studies reinforced the previous findings that anti-VEGF injections had remarkably improved the prognosis of CNV secondary to angioid streaks [12–14]. However, these studies only included ranibizumab as the first option of anti-VEGF treatment and one of these studies only included patients with PXE. Also, few results presented a follow-up of 60 months, and as far as we concern, there are no results at 72 months of follow-up. Moreover, these studies do not clarify whether the results may be generalized to all anti-VEGF and to all patients with CNV secondary to angioid streaks regardless of the associated systemic pathology.

Therefore, the primary endpoint of this study was to evaluate the effectiveness of intravitreal anti-VEGF injections at 48 months in the treatment of CNV associated with angioid streaks in a real-world study. The secondary endpoints were to evaluate the functional and structural changes after 60 and at 72 months of anti-VEGF treatment for those patients with longer follow-up.

## 2. Methods and Materials

### 2.1. Study Design

This is a multicenter retrospective cohort study, which included eyes with CNV secondary to angioid streaks treated with intravitreal anti-VEGF injections (with or without previous alternative treatments, such as laser photocoagulation or PDT). This study adhered to the tenets of the Declaration of Helsinki and was approved by the local Ethics Committee of five Portuguese hospitals.

All patients underwent an ophthalmologic examination, including measurement of best-corrected visual acuity (BCVA) using Early Treatment Diabetic Retinopathy Study (ETDRS) charts, slit-lamp biomicroscopy, fundus color photography, fluorescein angiography (FA), and spectral-domain optical coherence tomography (SD-OCT). The anti-VEGF treatments assessed in this study were ranibizumab (0.5 mg/0.05 mL), bevacizumab (1.25 mg/0.05 mL), and aflibercept (2 mg/0.05 mL). As patients were treated by the attending retina specialist in different institutions, different treatment agents and protocols were applied (fixed, Pro re nata—PRN, and “Treat and Extend”).

The presence of fibrosis or atrophy was assessed using SD-OCT.

### 2.2. Data Collected

We analyzed sociodemographic characteristics (age, gender), systemic associated disease conditions, previous treatments (photocoagulation laser, PDT), and CNV location (foveal, juxtafoveal, extrafoveal). BCVA (ETDRS letters); qualitative (atrophy and fibrosis) and quantitative (subfoveal thickness) SD-OCT parameters; and number of intravitreal injections were also collected at baseline (i.e., at the time of CNV diagnosis/treatment initiation) and at 3, 6, 12, 24, 36, 48, 60, and 72 months after initiating treatment.

### 2.3. Inclusion and Exclusion Criteria

Patients older than 18 years, treated with anti-VEGF agents for CNV secondary with angioid streaks at the retina departments from the previously referred Portuguese hospitals, were included. Patients had to have at least 36 months of follow-up data since the beginning of the anti-VEGF treatment. Eyes with prior intraocular surgery were also included. Eligible patients were identified by review of medical records. Both eyes of a patient were included for analysis, if eligible.

Patients were excluded if presented with any other ocular disease that could compromise vision in the study eye. Patients with systemic or ocular diseases that interfered with the acquisition of OCT were also excluded.

### 2.4. Primary Outcomes/Endpoints

The primary outcome was the variation of BCVA at 48 months of follow-up after initial anti-VEGF treatment.

### 2.5. Secondary Outcomes/Endpoints

The secondary outcomes were the variation of BCVA after 60 and at 72 months of anti-VEGF treatment (for those patients with longer follow-up), characterization of CNV location, variation of central macular thickness (CMT), development of complications (fibrosis/atrophy), and the number of anti-VEGF injections administered during follow-up.

### 2.6. Statistical Analysis

Statistical analysis was performed using the SPSS^®^ statistical software (version 26.0 for Windows; SPSS Inc., Chicago, IL, USA).

Quantitative variables were described through mean ± standard deviation and categorical variables through absolute (*n*) and relative (%) frequencies.

The Kolmogorov–Smirnov test and normal probability plots were used to confirm the normal distribution of continuous variables. Categorical variables were compared using a chi-squared test or, for low count variables, Fisher's exact test. Continuous independent variables following a normal distribution were compared using an independent-samples *t*-test, or a nonparametric Mann–Whitney *U*-test, whenever applicable, whereas continuous dependent variables were analyzed using a dependent-samples *t*-test or a Wilcoxon signed-rank test. Repeated-measures analysis of variance (ANOVA) was performed to analyze the changes in BCVA and CMT. Bonferroni's correction for multiple comparisons was applied. The association between two quantitative variables was assessed through Pearson's correlation coefficient (*r*) or Spearman's correlation coefficient (*r*_*s*_) in case the normality assumption was not verified.

Statistical significance for all the analyses was set at a *P* value less than 0.05.

As previously stated, both eyes from the same patient were included, which we considered an acceptable trade-off for a larger sample size of this rare condition.

## 3. Results

### 3.1. Characterization of the Patients

Thirty-nine eyes from 29 patients were included in the study. Demographic and baseline characteristics are described in Table 1. Excluding the 5 eyes previously treated with PDT, all the other eyes included were treatment naïve CNVs.

Overall, the mean study follow-up was 69.4 ± 34.5 months (range 36–135).

### 3.2. Functional Changes in Affected Eyes after Initiation of Anti-VEGF Treatment

**Figure 1:**
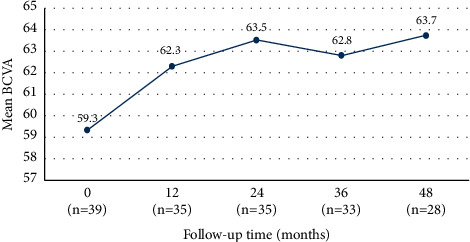
Best-corrected visual acuity (ETDRS letters) variation until 48 months (primary outcome). ^*∗*^*P* < 0.05.

The mean BCVA was 59.3 ± 23.3 letters at baseline and 63.7 ± 21.9 letters after 48 months (*P*=0.85) (Figure 1). At 3 months, a significant improvement in BCVA of 6.9 ± 11.7 letters was observed (*P*=0.003).

At baseline, 10 (25.6%) eyes had a BCVA inferior to 35 letters. After 48 months, 3 of 28 (10.7%) eyes had a BCVA inferior to 35 letters, 6 of 28 (21.4%) eyes showed a BCVA gain of ≥15 letters, and 12 of 28 (42.9%) had a stable BCVA (Table 2).

Baseline BCVA was significantly and negatively correlated with the difference between BCVA at 48 months and at baseline (*r*_s_ = −0.635; *P*=0.001). Furthermore, baseline BCVA was negatively correlated with baseline CMT (*r* = 0.514; *P*=0.004).

### 3.3. Anatomical Changes after Initiation of Anti-VEGF Treatment

**Figure 2:**
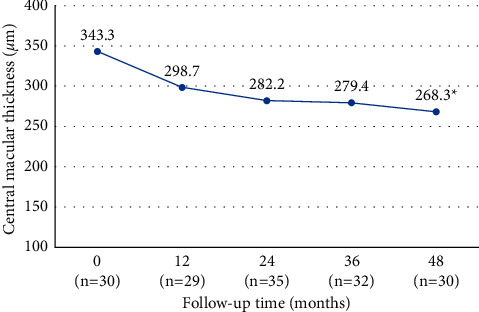
Mean central macular thickness (*μ*m) variation until 48 months (primary outcome). ^*∗*^*P* < 0.05.

The mean baseline CMT decreased from 343.3 ± 120.0 *μ*m to 318.8 ± 127.5 *μ*m at 3 months (*P*=0.091) and to 298.7 ± 83.8 *μ*m at 12 months (*P*=0.055). Although in the first 36 months, changes in CMT were not significant, at 48 months, CMT decreased significantly to 268.3 ± 65.4 *μ*m (*P*=0.021) compared with baseline (Figure 2). At 48 months, a CMT reduction superior to 10% was observed in 11 (45.8%) eyes, remained stable in 10 (41.7%) eyes, and increased more than 10% in 3 (12.5%) eyes. CMT variation during the first 48 months of follow-up is described in Table 3.

### 3.4. Number of Intravitreal Anti-VEGF Injections

**Figure 3:**
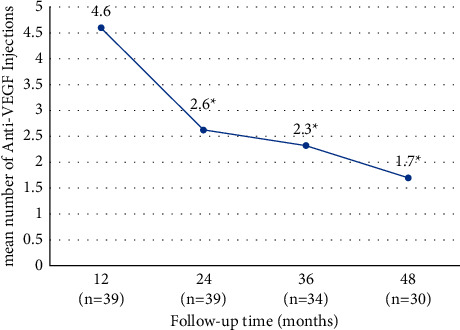
Mean number of injections per period until 48 months (primary outcome). The mean number of intravitreal injections was reduced significantly between 12 and 24 months (*P*=0.007), between 24 and 36 months (*P*=0.003), and between 36 and 48 months (*P*=0.002) compared with the number of injections performed in the first 12 months of follow-up.

The mean number of anti-VEGF injections was 4.6 ± 2.1 injections in the first 12 months (median 4, range 1–9), decreasing significantly across the study time points: 2.6 ± 2.9 injections (median 3, range 1–11) at 24 months (*P*=0.007 vs. 12 months); 2.3 ± 3.2 injections (median 2.5, range 1–17) at 36 months (*P*=0.003 vs. 24 months); and 1.7 ± 2.4 injections (median 2, range 1–8) at 48 months (*P*=0.002 vs. 36 months) (Figure 3). The total mean number of intravitreal injections administered at the end of the 48-month follow-up was 10.8 ± 9.0 (range 2–44). The mean number of injections received in the first 3 months was 2.2 injections. Sixteen of 39 (41%) eyes received loading dose, consisting of 3 injections in the first 3 months. No statistically significant association was found between eyes that received loading dose and BCVA at 48 months (*P*=0.746).

In a subgroup analysis, the affected eyes were divided according to the cumulative number of injections performed between baseline and 48 months: subgroup A—equal or less than 5 cumulative injections (*n* = 10), subgroup B—between 6 and 10 cumulative injections (*n* = 6), and subgroup C—equal or more than 11 cumulative injections (*n* = 10). No statistically significant associations were observed between any of the subgroups for cumulative number of injections and BCVA at 48 months. Moreover, in a univariate regression analysis, no statistically significant association was found between the number of injections performed and BCVA (*P*=0.093).

No systemic or ocular side effects were registered during the entire follow-up.

### 3.5. Complications during Follow-Up

Initially, 7 of 34 (20.6%) eyes had retinal atrophy and 6 of 35 (17.1%) eyes had fibrosis. At 48 months, 17 of 28 (60.7%) eyes had atrophy and 17 of 28 (60.7%) had fibrosis. At 48 months, foveal atrophy was statistically associated with inferior BCVA (*P*=0.047); however, foveal fibrosis was not associated with BCVA (*P*=0.647).

At 36 months, no statistically significant association was found between the CNV location and atrophy (*P*=0.412) or fibrosis development (*P*=0.509). Moreover, we did not observe a significant association between CMT and CNV location (*P*=0.628).

### 3.6. In the Subgroup of Patients with 60 and 72 Months of Follow-Up

Twenty (51.3%) eyes completed 60 months of follow-up. The mean BCVA was 59.0 ± 5.1 letters at baseline and 58.8 ± 5.6 letters at 60 months (*P*=0.974). The mean CMT was 361.5 ± 37.0 *μ*m at baseline and 243.5 ± 54.6 *μ*m at 60 months (*P*=0.018).

Fourteen (35.9%) eyes completed 72 months of follow-up. The mean BCVA was 52.8 ± 24.1 letters at baseline and 62.5 ± 21.9 letters at 72 months (*P*=0.060). The mean CMT was 347.7 ± 146.0 *μ*m at baseline and 231.0 ± 47.7 *μ*m after 72 months (*P*=0.200).

## 4. Discussion

Our real-world study suggested that treatment with anti-VEGF leads to a significant improvement in BCVA in the first 3 months, varying from 59.3 ± 23.3 ETDRS letters at baseline to 66.8 ± 19.5 letters at 3 months (*P*=0.003). After this time point, BCVA remained stable in 21 of 28 (75%) eyes at 48 months (Table 1).

The MINERVA study was the first phase III randomized controlled clinical trial to evaluate the efficacy and safety of ranibizumab 0.5 mg in patients with CNV associated with causes other than AMD and myopic CNV. In this study, 18 eyes with CNV secondary to angioid streaks were included. At 2 months, a treatment effect of 10 letters was observed with ranibizumab compared with sham in the CNV-angioid streaks [15], being similar to our results at 3 months.

In this study, patients had a mean age of 53.2 ± 12.1 years at the time of CNV diagnosis/treatment initiation, which is consistent with previous studies [12, 16, 17]. Moreover, the mean age of patients with PXE and angioid streaks complicated with CNV seems to be significantly lower than in patients with no PXE, 49.0 ± 10.5 years vs. 58.1 ± 12.4, *P*=0.023.

Tilleul et al. included 35 eyes with CNV secondary to angioid streaks treated with ranibizumab. At 4 years of follow-up, BCVA remained stabilized or improved in 22 of 35 eyes (62.9%). Macular thickness stabilized or decreased in 16 of 35 eyes (45.7%) [13]. In Tilleul et al. study, patients were older than the patients included in our study (mean age 63.7 ± 13.9 years [13] vs. 59.3 ± 23.3 years, respectively). The inferior results in BCVA obtained in Tilleul et al. study, compared with our study, could be explained partly by the older age of patients.

The PIXEL study included 98 eyes of 72 patients with PXE and CNV secondary to angioid streaks treated with ranibizumab [12]. The mean BCVA was 64.6 letters at baseline and 64.7 letters at 12 months, and it stabilized until the 4-year follow-up [12]. In our study, the mean BCVA was inferior at baseline, with 57.1 ± 23.8 letters compared with 64.6 letters in the PIXEL study. However, at 48 months, the mean BCVA in our study improved to 63.7 ± 21.4 letters, compared with 60.5 letters in PIXEL study. Therefore, our patients had an initial improvement in BCVA that was superior to PIXEL study and were able to maintain their BCVA values in the long term. One possible explanation for this observation was the fact that, in our study, patients had a lower BCVA at presentation and, thus, were expected to improve more than patients who had better BCVA at baseline. However, not all studies are consensual regarding the improvement of BCVA and its stability over time after anti-VEGF treatment. For instance, Giacomelli et al. observed a progressive deterioration in BCVA after treatment with ranibizumab or bevacizumab in PRN protocol [16]. Iacono et al. investigated the effects of intravitreal bevacizumab in 15 patients with non-subfoveal CNV secondary to angioid streaks and observed a statistically significant BCVA worsening at 3 years (65.8 ± 15.0 letters) compared with BCVA at 1 year (80.1 ± 5.4 letters) [18]. There are several factors that can lead to the different conclusions observed in the aforementioned studies, including CNV location, initial treatment delay, previous treatments (e.g., laser photocoagulations and PDT), and different treatment protocols (e.g., fixed, “treat and extend,” or PRN).

In the first 12 months, in our study, a mean number of 4.6 ± 2.1 injections were performed, being similar to the PIXEL study (4.1 ± 2.3 injections) [12], but inferior to Gliem et al.'s study (6.7 ± 2.6 injections) [19]. The total mean number of injections at the end of 48 months of follow-up was 10.8 ± 9.0 injections. In the Rosina et al.'s study, the median number of intravitreal injections was 2.5 (range 1–6) injections during a mean follow-up of 52 months. However, 8 (50%) eyes were previously treated with PDT (7 eyes) or argon laser (1 eye). The significant number of eyes previously treated with laser therapy could have biased in the results, explaining the lower number of injections needed in this study [20]. Finger et al. included 16 eyes treated with an average of 6.5 ± 5.7 injections over 28 months [17]. One possible explanation for the difference in the mean number of injections was the different regimen of treatment. As we stated before, as patients included in our study were treated in different institutions, by different retina specialists and over a long period of time, the treatment regimen was not uniform for all the patients. For instance, in Gliem et al.'s study, all patients received one initial injection of aflibercept, and further injections were based on monthly assessment of persistent or recurrent CNV activity [19]. Differently, other studies included in their sample eyes with fixed and PRN regimens, which might influence the final average number of injections [21]. Moreover, we would like to emphasize that, although the best timetable and criteria for injections have yet to be determined, the high recurrence of exudation in CNV proves the need for repeated injections. Unfortunately, there is still a great variability in access to intravitreal treatment as we can conclude for our study given the variance in the number of injections performed at 48 months. Similarly, other studies reported progressively less injections during the follow-up period [12, 13, 17, 18]. These observations are in agreement with the previous knowledge that inactivation of the CNV secondary to angioid streaks usually requires fewer injections than those associated with exudative age-related macular degeneration [13, 18].

Regarding the CMT variation, we observed a progressive reduction during the follow-up, as observed in other studies [12, 19, 20]. However, BCVA did not show a progressive improvement despite the decreasing trend in CMT, possibly due to RPE and photoreceptor damage with the long-standing of disease. It seems that, from the anatomic point of view, anti-VEGF agents are effective in reducing all the signs of CNV exudation, even though a morphologic recovery does not always correspond to a functional gain.

In 2006, the first favorable results for the treatment of CNV secondary to angioid streaks were achieved with bevacizumab [9]. Afterwards, several small studies about effectiveness of anti-VEGF in CNV secondary to angioid streaks were done with ranibizumab, bevacizumab, and aflibercept [17, 22–27]. Recently, Sekfali et al. concluded that switching from intravitreal ranibizumab to aflibercept represents a valid therapeutic option in patients with refractory or recurrent CNV secondary to angioid streaks [14]. In our study, we included patients treated with three anti-VEGF agents (i.e., ranibizumab, bevacizumab, and aflibercept). For all three anti-VEGF agents, a positive outcome for BCVA was reported over time, indicating that these treatments could be considered as the first-line therapy for CNV secondary to angioid streaks. However, as patients were treated in different institutions, by different retina specialists and over a long period of time, the treatment regimen was not uniform for all the patients. Therefore, taking into account the great variability in the treatment, not only in relation to the treatment regimen (fixed, PRN, treat, and extend) but also in relation to the type of drug administered, we were not able to perform a statistical analysis to obtain valid conclusions.

This study had important limitations. First, the observational design and the retrospective nature of the study were limitations as data from all patients were not available at each and every time point because some patients were not observed on the analyzed time points. Another drawback was related to the treatment with different anti-VEGF agents and the different treatment protocols. As this study included patients from different Portuguese institutions, the treatment regimen varied according to the clinical practice at each institution and according to the retinal specialist who assisted the patient. In addition, the anti-VEGF used, as well as the criteria for drug switch, varied between institutions and some patients were treated with different anti-VEGF agents over time. Thus, as previously referred, this study was not able to analyze the functional and structural outcomes caused by individual anti-VEGF agent or according to the treatment protocol. As CNV secondary to angioid streaks is a rare disorder, we included patients treated previously with PDT, which may bias the results.

Therefore, despite the limitations explained before, our study described the expected results in the real-world setting, contrary to many clinical trials that show promising results, but do not reflect the conditions of our daily clinical practice such as patients' difficulties to attend medical appointments and to comply with therapeutic regimens. Moreover, it presents favorable results that support the current clinical practice in Portugal and other European centers with anti-VEGF therapy for CNV related to angioid streaks.

Our study's strengths lie in its long-term follow-up, the significant number of patients included, and the fact that it describes the results of a real-life multicenter setting. To our knowledge, this is one of the studies with the longest follow-up time of patients with CNV secondary to angioid streaks performed so far.

In conclusion, this study demonstrated that the functional and morphologic response to anti-VEGF therapy for CNV related to angioid streaks is generally satisfactory and may be maintained on the long term. Of notice, the comparison across retrospective and real-world studies should be done with caution as the study populations vary with respect to the study settings, dosing regimens, previous treatment profiles, and underlying comorbidities. Further long-term prospective studies with a larger sample size are needed to evaluate the long-term effectiveness and the best protocol regimen of anti-VEGF treatment in CNV secondary to angioid streaks.

## Figures and Tables

**Table 1:** Demographic and baseline characteristics of patients with choroidal neovascularization secondary to angioid streaks.

	Mean ± std. deviation (relative frequency)
No. of patients included	29

No. of eyes included	39

Female	17 (58.6%)

Age (years)	53.0 ± 12.3

Both eyes affected	10 (34.5%)

PEX	15 (51.7%)

Age in PEX patients (years)	49.0 ± 10.5 (P=0.023)

PDT (eyes)	5 (12.8%)

CNV location	
Subfoveal	13 (33.3%)
Juxtafoveal	22 (56.4%)
Extrafoveal	4 (10.3%)

Baseline BCVA (ETDRS letters)	59.3 ± 23.3

Baseline CMT (*µ*m)	343.3 ± 120.0

Follow-up (months) (minimum-maximum)	69.4 ± 34.5 [36–135]

BCVA: best-corrected visual acuity, CMT: central macular thickness, CNV: choroidal neovascularization, ETDRS: Early Treatment Diabetic Retinopathy Study, PDT: photodynamic therapy, PEX: pseudoxanthoma elasticum.

**Table 2:** Best-corrected visual acuity variation (in ETDRS letters) by groups until 48 months (primary outcome).

ETDRS letter changes	12 months (*n* = 35)	24 months (*n* = 35)	36 months (*n* = 33)	48 months (*n* = 28)
≥ + 15 letters	8 (22.9%)	7 (20.0%)	7 (21.2%)	6 (21.4%)
+6 to + 14 letters	4 (11.4%)	5 (14.3%)	5 (15.2%)	3 (10.7%)
+5 to −5 letters	16 (45.7%)	15 (42.9%)	15 (45.5%)	12 (42.9%)
−6 to −14 letters	3 (8.6%)	3 (8.6%)	1 (3.0%)	3 (10.7%)
≤ − 15 letters	4 (11.4%)	5 (14.3%)	5 (15.2%)	4 (14.3%)

**Table 3:** Central macular thickness variation by groups until 48 months (primary outcome).

CMT variation	12 months (*n* = 25)	24 months (*n* = 28)	36 months (*n* = 25)	48 months (*n* = 22)
Decrease> − 50 *μ*m	7 (28.0%)	8 (28.6%)	7 (28.0%)	8 (36.4%)
Between −50 and + 50 *μ*m	15 (60.0%)	17 (60.7%)	15 (60.0%)	12 (54.5%)
Increase > + 50 *μ*m	3 (12.0%)	3 (10.7%)	3 (12.0%)	2 (9.1%)

## Data Availability

The data used to support the findings of this study are available from the corresponding author upon request.

## References

[1] Georgalas I., Papaconstantinou D., Koutsandrea C. (2008). Angioid streaks, clinical course, complications, and current therapeutic management. *Therapeutics and Clinical Risk Management*.

[2] Gliem M., Finger R. P., Fimmers R., Brinkmann C. K., Holz F. G., Charbel Issa P. (2013). Treatment of choroidal neovascularization due to angioid streaks: a comprehensive review. *Retina*.

[3] Brown D. M., Kaiser P. K., Michels M. (2006). Ranibizumab versus verteporfin for neovascular age-related macular degeneration. *New England Journal of Medicine*.

[4] Chatziralli I., Saitakis G., Dimitriou E. (2019). Angioid streaks: a comprehensive review from pathophysiology to treatment. *Retina*.

[5] Wecke T., Knop C., Schreiber W., Behrens-Baumann W. (2009). Intraocular injections of bevacizumab in rare indications-two cases. *Ophthalmologe, Der: Zeitschrift der Deutschen Ophthalmologischen Gesellschaft*.

[6] Chang L. K., Spaide R. F., Brue C., Freund K. B., Klancnik J. M., Slakter J. S. (2008). Bevacizumab treatment for subfoveal choroidal neovascularization from causes other than age-related macular degeneration. *Archives of Ophthalmology*.

[7] Bhatnagar P., Freund K. B., Spaide R. F. (2007). Intravitreal bevacizumab for the management of choroidal neovascularization in pseudoxanthoma elasticum. *Retina*.

[8] Sawa M., Gomi F., Tsujikawa M., Sakaguchi H., Tano Y. (2009). Long-term results of intravitreal bevacizumab injection for choroidal neovascularization secondary to angioid streaks. *American Journal of Ophthalmology*.

[9] Teixeira A., Moraes N., Farah M. E., Bonomo P. P. (2006). Choroidal neovascularization treated with intravitreal injection of bevacizumab (Avastin) in angioid streaks. *Acta Ophthalmologica Scandinavica*.

[10] Carneiro A. M., Silva R. M., Veludo M. J. (2011). Ranibizumab treatment for choroidal neovascularization from causes other than age-related macular degeneration and pathological myopia. *Ophthalmologica*.

[11] Parodi M. B., Cicinelli M. V., Marchese A. (2020). Intravitreal aflibercept for management of choroidal neovascularization secondary to angioid streaks: the Italian EYLEA-STRIE study. *European Journal of Ophthalmology*.

[12] Mimoun G., Ebran J. M., Grenet T., Donati A., Cohen S. Y., Ponthieux A. (2017). Ranibizumab for choroidal neovascularization secondary to pseudoxanthoma elasticum: 4-year results from the PIXEL study in France. *Graefes Archive for Clinical and Experimental Ophthalmology*.

[13] Tilleul J., Mimoun G., Querques G. (2016). Intravitreal ranibizumab for choroidal neovascularization in angioid streaks: four-year follow-up. *Retina*.

[14] Sekfali R., Mimoun G., Cohen S. Y. (2019). Switching from ranibizumab to aflibercept in choroidal neovascularization secondary to angioid streaks. *European Journal of Ophthalmology*.

[15] Lai T. Y. Y., Staurenghi G., Lanzetta P. (2018). Efficacy and safety of ranibizumab for the treatment of choroidal neovascularization due to uncommon cause: twelve-month results of the minerva study. *Retina*.

[16] Giacomelli G., Finocchio L., Biagini I. (2017). Long-term follow-up of choroidal neovascularization due to angioid streaks with pro re nata intravitreal anti-VEGF treatment. *Ophthalmologica*.

[17] Finger R. P., Charbel Issa P., Schmitz-Valckenberg S., Holz F. G., Scholl H. N. (2011). Long-term effectiveness of intravitreal bevacizumab for choroidal neovascularization secondary to angioid streaks in pseudoxanthoma elasticum. *Retina*.

[18] Iacono P., Battaglia Parodi M., La Spina C., Bandello F. (2016). Intravitreal bevacizumab for nonsubfoveal choroidal neovascularization associated with angioid streaks: 3-year follow-up study. *American Journal of Ophthalmology*.

[19] Gliem M., Birtel J., Herrmann P. (2020). Aflibercept for choroidal neovascularizations secondary to pseudoxanthoma elasticum: a prospective study. *Graefes Archive for Clinical and Experimental Ophthalmology*.

[20] Rosina C., Romano M., Cigada M., de Polo L., Staurenghi G., Bottoni F. (2015). Intravitreal bevacizumab for choroidal neovascularization secondary to angioid streaks: a long-term follow-up study. *European Journal of Ophthalmology*.

[21] Myung J. S., Bhatnagar P., Spaide R. F., Holz F. G., Scholl H. N. (2010). Long-term outcomes of intravitreal antivascular endothelial growth factor therapy for the management of choroidal neovascularization in pseudoxanthoma elasticum. *Retina*.

[22] Vaz-Pereira S., Collaco L., De Salvo G., van Zeller P. (2015). Intravitreal aflibercept for choroidal neovascularisation in angioid streaks. *Eye*.

[23] Esen E., Sizmaz S., Demircan N. (2015). Intravitreal aflibercept for management of subfoveal choroidal neovascularization secondary to angioid streaks. *Indian Journal of Ophthalmology*.

[24] Tetikoglu M., Sagdik H. M., Aktas S., Ozcura F. (2016). Intravitreal aflibercept for refractory choroidal neovascularization secondary to angioid streaks. *Eye*.

[25] El Matri L., Kort F., Bouraoui R., Karim B., Chebil A., Chaker N. (2011). Intravitreal bevacizumab for the treatment of choroidal neovascularization secondary to angioid streaks: one year of follow-up. *Acta Ophthalmologica*.

[26] Teixeira A., Mattos T., Velletri R. (2010). Clinical course of choroidal neovascularization secondary to angioid streaks treated with intravitreal bevacizumab. *Ophthalmic Surgery, Lasers and Imaging Retina*.

[27] Finger R. P., Charbel Issa P., Hendig D., Scholl H. P., Holz F. G. (2011). Monthly ranibizumab for choroidal neovascularizations secondary to angioid streaks in pseudoxanthoma elasticum: a one-year prospective study. *American Journal of Ophthalmology*.

